# Human monoclonal ScFv that bind to different functional domains of M2 and inhibit H5N1 influenza virus replication

**DOI:** 10.1186/1743-422X-10-148

**Published:** 2013-05-14

**Authors:** Tippawan Pissawong, Santi Maneewatch, Kanyarat Thueng-in, Potjanee Srimanote, Fonthip Dong-din-on, Jeeraphong Thanongsaksrikul, Thaweesak Songserm, Pongsri Tongtawe, Kunan Bangphoomi, Wanpen Chaicumpa

**Affiliations:** 1 Department of Parasitology, Graduate Program in Immunology, Mahidol University, Bangkok, 10700, Thailand; 2 Department of Molecular Tropical Medicine and Genetics, Mahidol University, Bangkok, 10400, Thailand; 3 Department of Microbiology and Immunology, Kasetsart University, Bangkok, 10900, Thailand; 4 Graduate Program in Biomedical Sciences, Thammasat University, Rangsit Campus, Pathumthani, 12120, Thailand; 5 Department of Biochemistry, Center for Agriculture Biotechnology, Kasetsart University, Bangkok, 10900, Thailand; 6 Department of Veterinary Pathology, Kasetsart University, Kam-paeng-saen Campus, Nakhopathom, 73140, Thailand; 7 Department of Biochemistry, Kasetsart University, Bangkok, 10900, Thailand; 8 Department of Parasitology, Laboratory for Research and Technology Development, Mahidol University, Bangkok 10700, Thailand

**Keywords:** Influenza virus, H5N1, M2 protein, Human ScFv, Phage display, Virus neutralization, Quantitative real-time RT-PCR, Mimotope, Molecular docking

## Abstract

**Background:**

Novel effective anti-influenza agent that tolerates influenza virus antigenic variation is needed. Highly conserved influenza virus M2 protein has multiple pivotal functions including ion channel activity for vRNP uncoating, anti-autophagy and virus assembly, morphogenesis and release. Thus, M2 is an attractive target of anti-influenza agents including small molecular drugs and specific antibodies.

**Methods:**

Fully human monoclonal single chain antibodies (HuScFv) specific to recombinant and native M2 proteins of A/H5N1 virus were produced from *huscfv*-phagemid transformed *E. coli* clones selected from a HuScFv phage display library using recombinant M2 of clade 1 A/H5N1 as panning antigen. The HuScFv were tested for their ability to inhibit replication of A/H5N1 of both homologous and heterologous clades. M2 domains bound by HuScFv of individual *E. coli* clones were identified by phage mimotope searching and computerized molecular docking.

**Results:**

HuScFv derived from four *huscfv*-phagemid transformed *E. coli* clones (no. 2, 19, 23 and 27) showed different amino acid sequences particularly at the CDRs. Cells infected with A/H5N1 influenza viruses (both adamantane sensitive and resistant) that had been exposed to the HuScFv had reduced virus release and intracellular virus. Phage peptide mimotope search and multiple alignments revealed that conformational epitopes of HuScFv2 located at the residues important for ion channel activity, anti-autophagy and M1 binding; epitopic residues of HuScFv19 located at the M2 amphipathic helix and cytoplasmic tail important for anti-autophagy, virus assembly, morphogenesis and release; epitope of HuScFv23 involved residues important for the M2 activities similar to HuScFv2 and also amphipathic helix residues for viral budding and release while HuScFv27 epitope spanned ectodomain, ion channel and anti-autophagy residues. Results of computerized homology modelling and molecular docking conformed to the epitope identification by phages.

**Conclusions:**

HuScFv that bound to highly conserved epitopes across influenza A subtypes and human pathogenic H5N1clades located on different functional domains of M2 were produced. The HuScFv reduced viral release and intracellular virus of infected cells. While the molecular mechanisms of the HuScFv await experimental validation, the small human antibody fragments have high potential for developing further as a safe, novel and mutation tolerable anti-influenza agent especially against drug resistant variants.

## Background

Influenza virus matrix-2 (M2) is a type III transmembrane protein of a class IA viroporin family [1]. Each M2 molecule comprises different domains: N-terminal ectodomain (M2e; 25 residues; 1–25), transmembrane domain (21 residues; 26–46), amphipathic helix (16 residues; 47–62) and C-terminal (35 residues; 63–97) [2,3]. M2 homotetrameric molecules are present on infected cell surface (~ two M2 tetramers per one HA trimer) and virion (~23-60 M2 tetramers per a virion) [4,5]. At the early phase of infection, the protein functions as a pH-activated ion channel allowing protons to enter the virion causing release of the vRNPs from the endosome into cytoplasm for further replication in nucleus [6]. Consequently, M2 prevents acid-induced conformational change of the newly synthesized hemagglutinin molecules that are cleaved in trans-Golgi network [7]. Recently, M2 was found to block fusion of autophagosomes to lysosomes and inhibit autophagy causing accumulation of the autophagosomes which compromised virus infected cell survival [8]. At the late replication process, M2 is recruited by M1 to the site of virus budding for virion assembly and morphogenesis [3]. Thereafter, M2 amphipathic helix alters membrane curvature at the neck of the budding virion causing membrane scission and the virus release [3]. Because of the multiple pivotal functions in the influenza virus infectious cycle, M2 has been an attractive target of anti-influenza agents.

Presently, there are two families of anti-influenza drugs: adamantanes that inhibit M2 ion channel activity and neuraminidase inhibitors [9]. These drugs must be taken at the early phase of infection for high therapeutic effectiveness [10]. Drug resistant virus mutants have emerged continuously causing treatment failure [11]. Adamantane frequently causes side effect on the central nervous system [12]. There is a need of novel anti-influenza agent that is safe and more tolerable to the virus mutations than the currently available drugs.

Antibodies have been used with success for influenza treatment [13-19]. Convalescing plasma could rescue patient infected with drug escape H5N1 virus mutant [17]. Human ScFv specific to H5 could protect mice from lethal challenges with H5N1 viruses of both clades 1 and 2 [18]. Passively transferred M2e-specific monoclonal antibody to mice caused acceleration of the lung viral clearance [19]. Antibody is more tolerable to mutation of the viral target than small molecular drugs [20]. Thus in this study, monoclonal single chain antibody fragments (HuScFv) which are fully human proteins and bound specifically to recombinant and native M2 proteins of H5N1 viruses were produced. The HuScFv interfered with the virus replication. Presumptive epitopes of the effective HuScFv on the M2 monomer were identified by means of phage mimotopic peptide searching and multiple alignments. Homology modelling and molecular docking were used to predict the regions and residues of the tetrameric M2 ion channel bound by the small antibodies in order to predict the molecular mechanisms of the antibody in mediating inhibition of the virus replication.

## Results

### M2 specific HuScFv and characteristics

**Figure 1:**
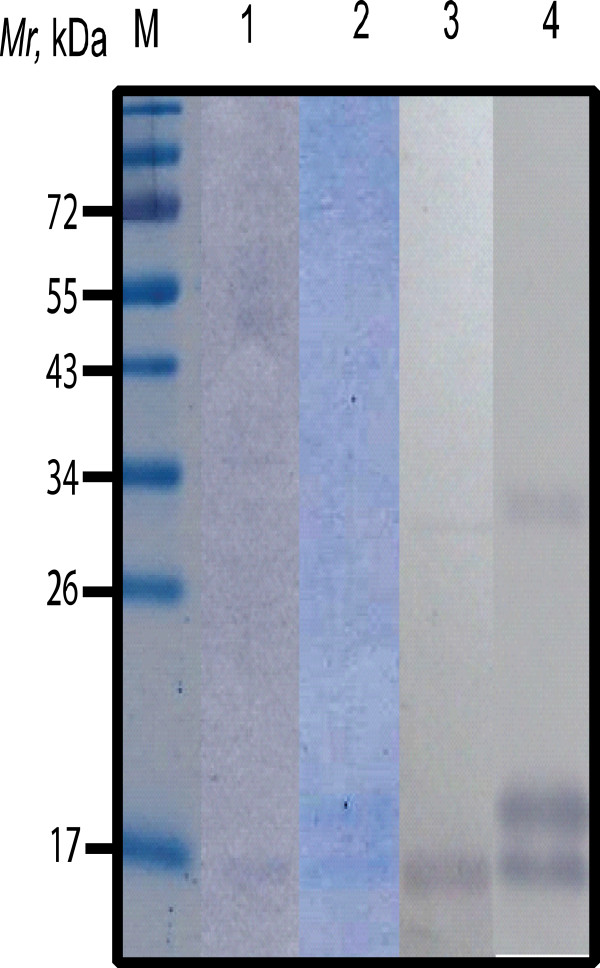
**SDS-PAGE and Western blot patterns of recombinant M2 protein. **Lane M: protein molecular weight marker; lanes 1 and 2: SDS-PAGE patterns of the soluble rM2 and refolded rM2 from *E. coli *inclusion, respectively. Soluble rM2 appears as a band of 17 kDa while M2 prepared from the *E. coli *inclusion appears as a protein doublet which the lower band is mature rM2 and the upper band is rM2 linked with pelB1 leader peptide of pET20b(+) (21 kDa). Lanes 3 and 4: Western blot patterns of the soluble rM2 and pelB1-linked M2, respectively. For the Western blotting, strips of nitrocellulose membrane (NC) blotted with SDS-PAGE separated-rM2 were incubated with mouse anti-6x-histidine antibody, goat anti-mouse immunoglobulin-alkaline phosphatase conjugate and BCIP/NBT substrate, respective. Uppermost band in lane 4 (arrow) is rM2 dimer. Numbers at the left are relative molecular masses (*Mr*) of proteins.

Recombinant M2 (rM2) of A/duck/Thailand/144/2005 (H5N1) (clade 1) was successfully purified from lysate of transformed *E. coli* carrying pET-20b(+) vector with full length *M2* cDNA insert. The protein was verified by SDS-PAGE and Western blotting (Figure 1). The deduced amino acid sequence of the cloned *M2* showed 100% homology to the M2 sequences of various H5N1 isolates (data not shown) [21].

**Figure 2:**
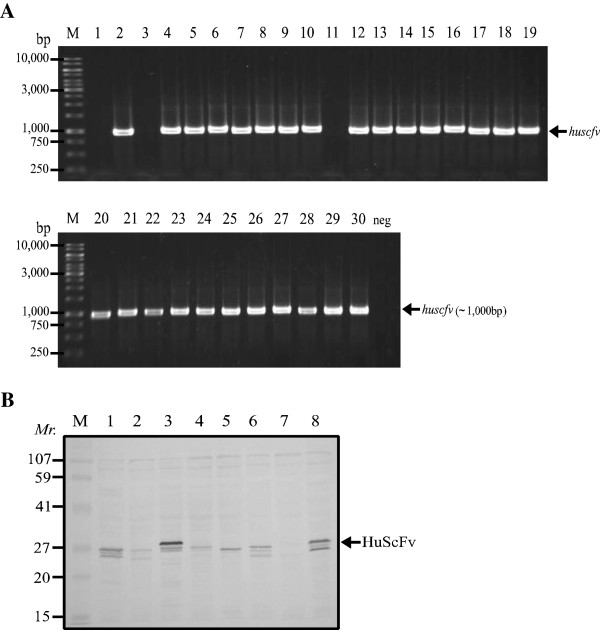
***E. coli *clones carrying *huscfv *and HuScFv in their lysates revealed by Western blot analysis. **(**A**) Lane M: One kb DNA ladder; lanes 2, 4–10 and 12–30: amplicons of *huscfv* in *E. coli *clones 2, 4–10 and 12–30, respectively; lane neg, negative control which was PCR result of PCR Master mix without DNA template. Numbers at the left of both blocks are DNA sizes in base pairs (bp). (**B**) Western blot results for detecting HuScFv in lysates of 8 representative *huscfv* positive clones. Lane M: protein standard marker; lanes 1–6 and 8: positive HuScFv (~27 kDa) in lysates of *huscfv *positive *E. coli *clones; lane 7: lysate of *huscfv *positive *E. coli *that did not express HuScFv. Numbers at the left are relative molecular masses (*Mr*) of proteins.

**Figure 3:**
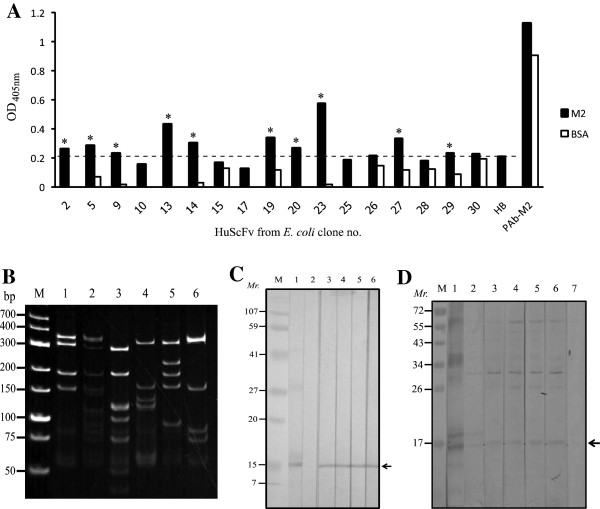
**Characterization of the selected phagemid transformed-HB2151 *E. coli *clones. **(**A**) Indirect ELISA results showing the binding of HuScFv in lysates of 17 *huscfv *positive *E. coli *clones to rM2 and BSA control. HuScFv of 10 clones (no. 2, 5, 9, 13, 14, 19, 20, 23, 27 and 29) bound specifically to the rM2. (**B**) RFLP of *huscfv *sequences of the 10 ELISA positive clones showed 6 different DNA patterns. Lanes 1–6: RFLP of clones 2, 5, 14, 19, 23 and 27, respectively. (**C**) Western blot patterns of HuScFv-2, -19, -23 and -27 that bound to rM2; HuScFv-5 and -14 did not bind to the rM2 (data not shown). Lane M: protein molecular weight standard; lane 1: positive control (rM2 blotted strip was probed with mouse PAb to rM2); lane 2: negative control (rM2 blot was probed with lysate of normal *E. coli*); lanes 3–6, rM2 blotted strips probed with HuScFv-2, -19, -23, and -27, respectively. The antigen-antibody reactive bands were revealed by using mouse anti-E tag-enzyme conjugate and substrate. (**D**) Western blot results for determining the binding of HuScFv of clones 2, 19, 23 and 27 to nM2. Lane M: protein molecular weight standard; lane 1: positive control which the SDS-PAGE separated rM2 purified from the *E. coli *lysate was probed with mouse PAb to rM2; lane 2: positive control which SDS-PAGE separated H5N1 virus lysate containing nM2 was probed with mouse PAb to rM2; lanes 3–6, nM2 blotted strips probed with HuScFv of clones no. 2, 19, 23, and 27, respectively; the antigen-antibody reactive bands were detected by mouse anti-6x His tag; lane 7: negative control which the nM2 blotted strip was probed with lysate of normal *E. coli*. Numbers at the left of (**C**) and (**D**) are relative molecular masses (*Mr*) of proteins.

The recombinant M2 (rM2) was used as antigen in single round phage panning for selecting the antigen bound phage clones. After infecting HB2151 *E. coli* with the rM2 bound-phages, 30 *E. coli* clones were randomly selected and screened for the presence of gene sequence coding for HuScFv (*huscfv*) and 27 clones (90%) were positive (Figure 2A). Among the *huscfv* positive *E. coli* clones, 17 clones could express HuScFv (57%) as determined by Western blotting. Figure 2B shows Western blot patterns of HuScFv in lysates of 7 representative *huscfv* positive *E. coli* clones. HuScFv in lysates of 10/17 *E. coli* clones (no. 2, 5, 9, 13, 14, 19, 20, 23, 27 and 29) gave significant binding to the rM2 by indirect ELISA (Figure 3A). They also bound to native M2 (nM2) in homogenate of clade 2 A/H5N1 (A/chicken/Thailand/NP-172/2006) infected cells by Western blotting (data not shown). The *huscfv* sequences of the 10 *E. coli* clones showed 6 different DNA banding patterns after *Mva*I digestion, 14% SDS-PAGE and ethidium bromide staining (Figure 3B). Clones no. 5 and 20 had pattern 2 (lane 2); clones no. 9 and 14 had pattern 3 (lane 3), clones no. 13 and 23 had pattern 5 (lane 5) and clones no. 2, 19 and 27 had patterns 1, 4 and 6, respectively (lanes 1, 4 and 6). Western blot analysis showed that HuScFv of clones no. 2, 19, 23 and 27 bound to rM2 and nM2 (Figure 3C and 3D, respectively**)**. They also bound to nM2 in the virus infected cells as tested by immunfluorescence assay (Additional file 1: Figure S1). Deduced amino acid sequences of *huscfv* of the four clones showed high diversity especially at the complementarity determining regions (CDRs 1–3) of both VH and VL chains (data not shown) indicating their different epitope specificities.

### M2 specific-HuScFv mediated interference of the influenza virus replication

**Figure 4:**
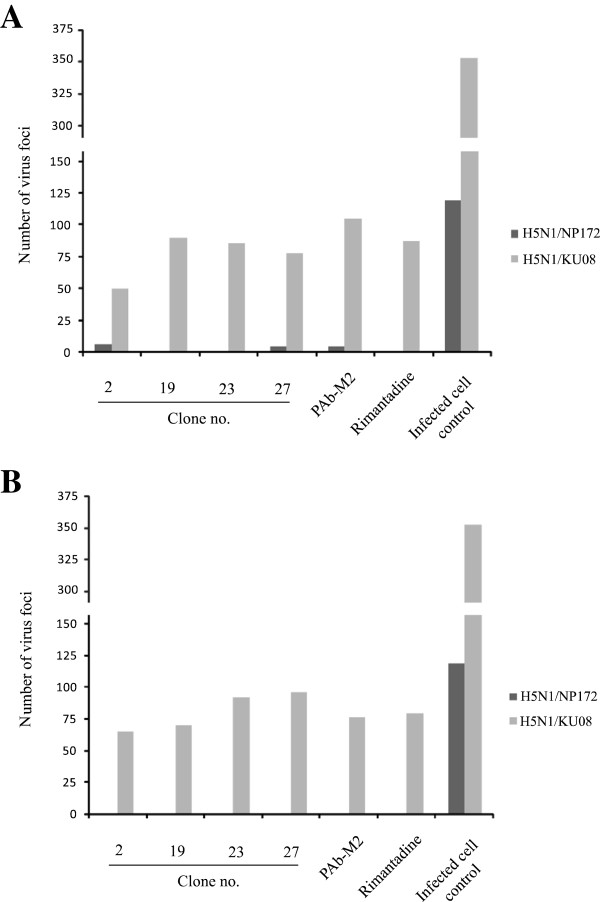
**Numbers of virus foci in the MDCK cells infected with adamantane sensitive and resistant viruses. **Figure 4**A **show results of experiment 1 which the viruses were added directly to the MDCK cell monolayer and the infected cells were cultured in the medium containing HuScFv, PAb to M2, rimantadine or medium alone for 15 h. No foci of the rimantadine sensitive virus were found in the infected cells cultured with HuScFv of clones no. 19 and 23 and rimantadine while only few foci were seen in cells exposed to HuScFv of clones no. 2 and 27 and PAb. The numbers of amantadine-resistant virus foci in infected cells cultured in HuScFv, PAb to M2 and rimantadine supplemented medium were markedly reduced compared with the infected cell control. Figure 4**B **shows results of experiment 2 which the viruses had been exposed to M2 specific-HuScFv, PAb to M2, rimatadine or medium before adding to the cell monolayer and the infected cells were cultured in the respective inhibitors or medium alone, the adamantane sensitive virus foci were not found in the cells exposed to all inhibitors. Numbers of foci of the amantadine resistant virus treated with the HuScFv, PAb to M2 and rimantadine were markedly reduced compared with the infected cell control.

Figure 4A shows numbers of virus foci in MDCK cells infected directly with adamantane sensitive and resistant viruses [A/chicken/Thailand/NP-172/2006 (H5N1) clade 2 and A/dog/Thailand/KU-08/2004 (H5N1) clade 1, respectively] and the cells were grown in medium containing HuScFv, PAb, rimantadine or medium alone for 15 h (see experiment 1 in Methods ). No foci of the rimantadine sensitive virus were found in the infected cells cultured with HuScFv of clones no. 19 and 23 and rimantadine while only few foci were seen in cells exposed to HuScFv of clones no. 2 and 27 and PAb. The numbers of amantadine-resistant virus foci in infected cells cultured in HuScFv, PAb to M2 and rimantadine supplemented medium were markedly reduced compared with the infected cell control. For experiment 2 which the viruses had been exposed to M2 specific-HuScFv, PAb to M2, rimatadine or medium before adding to the cell monolayer and the infected cells were cultured in the respective inhibitors or medium alone, the adamantane sensitive virus foci were not found in the cells exposed to all inhibitors. Numbers of foci of the amantadine resistant virus treated with the HuScFv, PAb to M2 and rimantadine were markedly reduced compared with the infected cell control (Figure 4B). Additional file 2: Figure S2 shows appearances of the virus foci in MDCK cells of Figure 4B.

**Figure 5:**
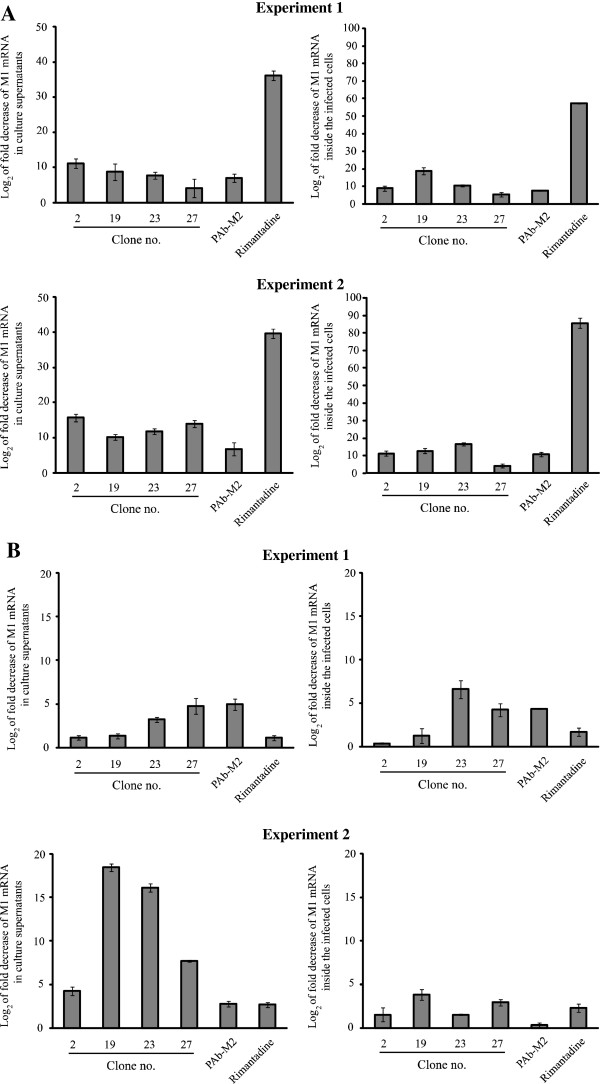
**Log**_**2 **_**of fold decrease in M1 mRNA in culture supernatants and inside the MDCK cells infected with amantadine sensitive and resistant influenza viruses**. Figure 5**A **and 5**B **show results of various inhibitors including HuScFv/rimantadine/PAb and medium control on amantadine sensitive virus [A/chicken/Thailand/NP172/2006 (H5N1) (clade 2)] and resistant virus [A/dog/Thailand/KU08/2004 (H5N1) (clade 1)], respectively.

Figure 5 shows qPCR results of M1 mRNA in culture supernatants and inside the cells that had been infected with adamantane sensitive and resistant influenza viruses, i.e., A/chicken/Thailand/NP172/2006 (H5N1) (clade 2.3.4) and A/dog/Thailand/KU08/2004 (H5N1) (clade 1), respectively. The viruses were added directly to the cells (experiment 1) or mixed with HuScFv, PAb to M2, rimantadine or medium alone before adding to cells (experiment 2). After allowing cellular entry and removing extracellular viruses, the infected cells were cultured in the presence of HuScFv/PAb/rimantadine or medium alone for 15 h. In Figure 5A, the amantadine sensitive virus was used to infect the cells. From both experiments, rimantadine was the best in reducing the viral M1 mRNA amounts in both supernatants and inside the cells. The HuScFv of all clones and PAb could reduce significantly the virus in both culture supernatants and inside the cells in comparison with the infected cells (*p* < 0.05). In experiment 1 of drug resistant virus (Figure 5B), rimantadine was less effective than the HuScFv of clones no. 23 and 27 and PAb in reducing the M1 mRNA in the culture supernatants and equally effective to the HuScFv of clones 2 and 19. The effectiveness in reducing intracellular virus, in increasing order of magnitude, were HuScFv2 < HuScFv19 < rimantadine < HuScFv27 = PAb < HuScFv23. For experiment 2, the effectiveness of the inhibitors in reducing the virus release were rimantadine = PAb < HuScFv2 < HuScFv27 < HuScFv23 < HuScFv19. For reduction of intracellular virus, HuScFv of clones no. 19 and 27 were most effective.

### Phage peptides that bound to HuScFv (mimotopes), HuScFv epitopes on M2 and validation of the phage mimotopes

**Figure 6:**
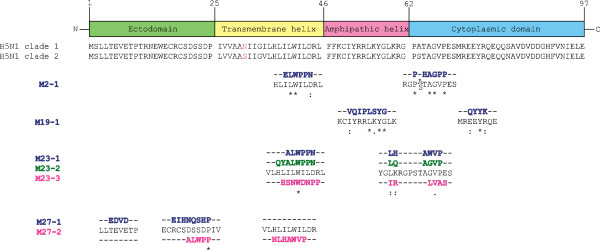
**Phage peptide sequences matched with residues of A/H5N1 monomeric M2 molecule (epitopes of HuScFv). **HuScFv2 mimotope type (M2-1: ELWPPNPHAGPP) matched with amino acid residues in transmembrane helix and cytoplasmic C-terminal of M2 of both viruses; HuScFv19 mimotope type (M19-1: VQIPLSYGQYYK) matched with residues of the M2 amphipathic helix and cytoplasmic C-terminal; HuScFv23 mimotope types (M23-1:ALWPPNLHAWVP, M23-2: QYALWPPNLQAGVP, M23-3: HSNWDNPPIRLVAS) matched with residues in transmembrane domain, amphipathic helix and cytoplasmic C-terminal; mimotope types of HuScFv27 (M27-: EDVDEIHNQSHP and M27-2: ALWPPNLHANVP) matched with M2 residues in ectodomain and transmembrane helix.

Ten clones each of phages displaying 12 mer peptides that bound to HuScFv- 2, -19, -23 and -27 were sequences (see Methods). All 10 clones that bound to HuScFv2 revealed identical peptide sequence (ELWPPNPHAGPP) designated mimotope type M2-1. There was also one mimotope type, i.e., M19-1 (VQIPLSYGQYYK) of HuScFv19. HuScFv23 had three mimotope types: M23-1 (ALWPPNLHAWVP), M23-2 (QYALWPPNLQAGVP) and M23-3 (HSNWDMPPIRLVAS). Two mimotope types were deduced from 10 HuScFv27 bound phage clones: M27-1 (EDVDEIHNQSHP) and M27-2 (ALWPPNLHAWVP). Sequences of all mimotope types were aligned with the monomeric M2 sequences of A/H5N1 of the database, i.e., clade 1: AY651385.1 and clade 2: AB478035.1, in order to locate tentative regions and residues on the M2 bound by the HuScFv (epitopes) (Figure 6). The 12 residue phage mimotope peptide of HuScFv of clone no. 2 (M2-1) matched with 39ILWILD44 in transmembrane domain and 63P-TAGVP69 in cytoplasmic domain of M2 of both clades. The mimotope M19-1 matched with 51IYRRLKYG58 of amphipathic helix and 74EEYR77 of cytoplasmic domain. The M23-1 matched with residues 39ILWILD44 of transmembrane helix and 59LK60 of amphipathic helix and 66AGVP69 of cytoplasmic doamin; M23-2 matched with 37HLILWILD44 of transmembrane helix, 59LK60 of amphipathic helix and 66AGVP69 of cytoplasmic domain and M23-3 matched with 38LILWILDR45 of transmembrane helix, 59LK60 of amphipathic helix and 67GVPE70 of cytoplasmic domain. The mimotope M27-1 matched with 5TEVE8 and 18RCSDSSDP25 in ectodomain and M27-2: matched with 21DSSDP26 in ectodomain and 37HLILWIL43 in transmembrane helix.

**Figure 7:**
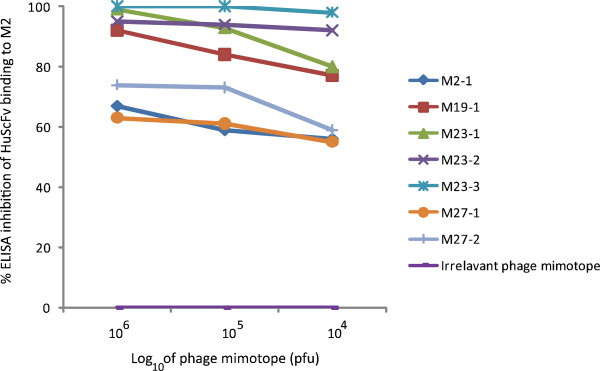
**Competitive ELISA for determining efficiencies of phage mimotopes in blocking the HuScFv binding to rM2. **In the assay, individual phage mimotope types, i.e., M2-1: ALWPPNLHAWVP, M19-1: VQIPLSYGQYYK, M23-1: HSNWDMPPIRLVAS, M23-2: QYALWPPNQAGVP, and M23-3: HSNWDMPPIRLVAS, M27-1: EDVDEIHNQSHP and M27-2: ALWPPNLHAWVP at 10^4^, 10^5 ^and 10^6 ^pfu were mixed with individual HuScFv (5 μg) before adding to the ELISA well containing immobilized rM2 (10 μg). HuScFv mixed with irrelevant phage mimotope served as background inhibition controls. The percent ELISA inhibition was calculated. The results indicated that the mimotopes could inhibit the HuScFv binding to rM2; implying that they carried the amino acid residues analogous to the native M2 residues that could be bound by the respective HuScFv.

Competitive ELISA for determining the ability of the phage mimotopes and irrelevant phage mimotope in inhibiting the HuScFv binding to the rM2 are shown in Figure 7. Binding of the HuScFv clones no. 2, 19, 23, and 27 to the M2 was partially inhibited by the phage mimotope types. The percent inhibition was not 100% for all HuScFv due to binding of the phage peptides to only partial regions of M2. Nevertheless, the results indicated that the mimotopes carried the amino acid residues analogous to the native M2 which verified the mimotope search data.

**Figure 8:**
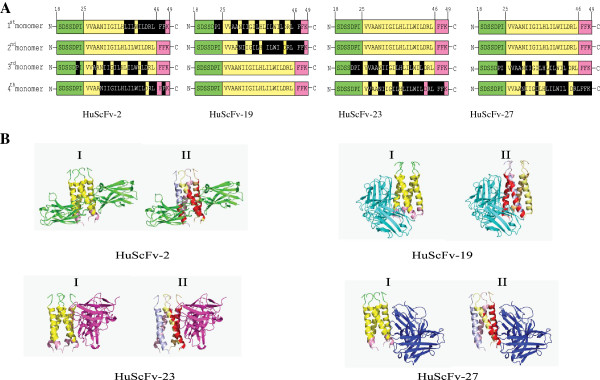
**Residues and areas of individual monomers of tetrameric M2 ion channel interacted with the HuScFv. **(**A**) Predicted M2 residues (black shades) in individual monomers of the ion channel which were bound by the HuScFv. (**B**) Results of molecular docking between the HuScFv with the tetrameric M2 ion channel template which was obtained from PDB entry 2LY0. (I) Interaction of HuScFv-2 (green), -19 (cyan), -23 (violet) and -27 (blue) with tetrameric M2 ion channel template. The so-obtained template contained only six residues of ectodomain (green), complete transmembrane helix (yellow) and 3 residues of amphipathic helix (pink) but lacked completely the C-terminal residues. (II) Regions (red shades) of individual M2 monomers (colored in light blue, light pink, light brown and pale yellow for monomers 1–4, respectively) that interacted with the HuScFv.

Computerized binding between HuScFv and tetrameric M2 ion channel are shown in Figure 8. The RDOCK and interactive M2 residues for all HuScFv are shown in Figure 8A. The RDOCK between the HuScFv2 and the tetrameric M2 ion channel was -30.38; the HuScFv2 bound to residues 38LIL-ILDRLF47 of the first monomer, residues 25P---A--II-IL--ILW-LD44 of the third monomer and residues 31NIIGILHLILWIL-RL-F48 of the fourth monomer. The RDOCK between HuScFv19 and the M2 tetramer was also -30.48; the HuScFv19 bound to 25PI---AA-II--L--IL--L--LF48 of the first monomer and 31NI---IL-LILWIL-RLFF49 of the second monomer. The RDOCK between the HuScFv23 and the M2 tetramer was -21.99; the HuScFv23 bound to residues 24DPI-VAA-II--L--L----L44 of the third monomer and 28V--NI--IL-LILWIL-RLFF49 of the fourth monomer. The RDOCK between the HuScFv27 and the M2 tetramer was -27.92; the HuScFv27 bound to residues 25PI-VAA-II--LH-IL--L43 of the third monomer and 31N---LILWIL-LFFK50 of the fourth monomer.

Regions of individual monomers of the tetrameric M2 ion channel template obtained from PDB entry 2LY0 that interacted with the HuScFv-2, -19, -23 and -27 were obtained from computerized molecular docking as shown in Figure 8B. The so-obtained structure of this PDB entry contained only six residues of ectodomain, complete transmembrane helix and 3 residues of amphipathic helix but lacks completely the C-terminal residues.

### Multiple alignments of M2 amino acid sequences of various influenza viruses

Influenza A viruses belonging to different subtypes and human infected H5N1 clades revealed highly conserved M2 sequences especially at the HuScFv binding sites (Additional file 3: Figure S3).

## Discussions

The surface exposed M2 protein of influenza viruses has several pivotal functions in the virus infectious cycle. Therefore, the protein has been attractive target of anti-influenza agents. In this study, fully human single chain antibody variable fragments (HuScFv) that bound specifically to recombinant and native M2 of A/H5N1 viruses belonging to different clades were successfully produced using phage display technology. The so-produced small antibodies were tested for their ability to inhibit the virus replication using rimantadine and polyclonal antibodies to full length recombinant M2 (PAb) as positive inhibitors. As expected, rimantadine was highly effective for the drug sensitive virus variant and rather refractory for the resistant mutant. The observed ability of the PAb in reducing the viruses inside the cells and in cell culture supernatant should be due to viral aggregation and steric hindrance of the ion channel activity. The HuScFv derived from all four *E. coli* clones also caused significant reduction of the amounts of both drug sensitive and resistant A/H5N1 viruses in the infected cells and the culture supernatants.

The first known M2 function is the pH dependent selective proton channel activity formed by homotetrameic M2 molecules on the virus surface. The channel is important for vRNP uncoating from endosome into cytoplasm and subsequent replication in nucleus [6]. The ion channel pore is lined by the polar amino acids Val^27^, Ser^31^, Gly^34^, His^37^, Trp^41^, Asp^44^ and Arg^45^ of the transmembrane (TM) tetrahelices while the channel integrity is maintained by TM non-polar residues and the positively charged residues of the TM and the amphipathic helix [22]. At the high pH, the pore is closed by the TM helices and the constrictive gates mediated by Val^27^ at the N-terminal ion entrance and Trp^41^ at the C-terminal ion exit [2,22]. Under endosomal low pH condition, the highly proton selective His^37^ senses the acidification at the N-terminal and allows inward flow of H^+^ through the channel, whereas the gate formed by linking the Trp^41^ indole ring side chain with Asp^44^ and Arg^45^ is open; thus allowing the outward flow of the proton to the C-terminal and release [23]. Adamantane compounds including amantadine and its derivative rimantadine block the ion channel activity of influenza A viruses. Amantadine obstructs the ion channel pore by binding to Ser^31^ and the surrounding Val^27^, Ala^30^ and Gly^34^[24] while rimantadine binds to the gate at a lipid-facing pocket of the channel formed by Trp^41^, Ile^42^, and Arg^45^ from one TM helix and Leu^40^, Leu^43^, and Asp^44^ of the nearby helix [2]. Resistance to the drugs has occurred in > 98% of transmissible A/H1N1, A/H5N1 and A/H3N2 strains by mutations, most frequently S31N and less so V27A and L26F [25,26]. The mutations cause failure of ion channel blocking by amantadine and ineffective fitting of rimantadine into the channel pocket due to the weakness of the TM helix packing [27]. Several compounds that are potent inhibitors of V27A and L26F mutants have been produced and tested [28]. However, effective inhibitor of S31N mutant has not been found [29].

It is known that during various virus infections, such as hepatitis C, human immunodeficiency, polio, toga and influenza, cellular membrane has increased permeability [30-35]. Intracellular entry of HuScFv specific to influenza virus protein and co-localization of the antibody with the specific protein target inside the infected cells have been observed by confocal microscopy (data not shown). In this study, the monovalent HuScFv which devoid of virus agglutinating ability were effective in inhibiting the virus replication indicating that the small antibodies could enter the virus infected cells and exerted M2 function interference. Epitopes of the HuScFv identified by means of phage mimotope searching and mimotope inhibition ELISA as well as molecular docking pointed out that the HuScFv of clones no. 2, 23, and 27 bound to the gate area (Trp^41^) of the M2 ion channel. Therefore, the observed ability of the HuScFv of these clones in inhibiting the influenza virus replication was likely to be a consequence of the antibody mediated interference of the ion channel activity leading to failure of the virus uncoating. Moreover, the pH gradient equilibration between the trans-Golgi network and cytosol by the ion channel activity could be interrupted also, causing pre-mature maturation of the virus hemagglutinin [7]. The amphipathic helix and cytoplasmic domain of the influenza virus M2 induces cellular membrane curvature during the virus assembly, increases vRNP packaging by M1-M2 interaction and assists the membrane scission in virus budding process [3]. The finding that peptide epitopes of the HuScFv of clones no. 2, 19 and 23 located in the amphipathic and cytoplasmic domains of the M2 indicated that the antibodies might inhibit the virus replication by interfering with the functions of the amphipathic helix and the cytoplasmic tail. During the infection, the N-terminal 1–60 residues of the influenza virus M2 compromise survival of influenza virus-infected cells by inhibiting the cellular macroautophagy formation [8]. The finding that many epitopic residues of M2 potentially bound by the HuScFv of all clones also located in the N-terminal portion indicating that the antibodies might as well counteract the M2 function on anti-autophagosome-lysosome fusion. Multiple alignments of the M2 amino acid sequences of various influenza A subtypes and human pathogenic clades of subtype H5N1 revealed that the HuScFv epitopic peptides are highly conserved implying that the HuScFv should be able to counteract the M2 activities across subtypes and H5N1 clades. Although the speculated molecular mechanisms of the HuScFv produced in this study in interfering with the influenza virus replication await experimental verification, the HuScFv have high potential for developing further as a safe, novel and mutation tolerable pan anti-influenza agent.

## Conclusions

Human monoclonal single chain antibody variable fragments (HuScFv) that bound specifically to both recombinant and native M2 proteins of A/H5N1 influenza viruses were produced by using phage display technology. HuScFv from four phagemid transformed *E. coli* clones mediated replication inhibition of H5N1 influenza viruses belonging to clades 1 and 2. Phage mimotope searching and computerized homology modeling and molecular docking revealed that the HuScFv of the four bacterial clones reacted with different functional domains of the influenza virus M2. The small human antibody fragments have high potential for developing further as a safe, novel and mutation tolerable broad spectrum anti-influenza agent.

## Methods

### Influenza viruses

Influenza A virus strains used in this study are listed in Table 1. They were propagated in 8–10 day old embryonated chicken eggs. The allantoic fluids of all eggs were collected, pooled, filtrated through 0.2 μM membrane (Pall, USA) and kept in small aliquots at -80°C. Virus titers were determined by hemagglutination (HA) assay against 1% chicken erythrocytes [36]. Optimal multiplicity of infection (MOI) was calculated from titration of 50% tissue culture infected dose (TCID50) [36,37] in Mardin-Darby canine kidney (MDCK) cells grown at 37°C under 5% CO_2_ atmosphere in Dulbecco’s modified Eagle’s medium (DMEM) (Invitrogen, USA) supplemented with 2% heat inactivated fetal bovine serum (FBS) (Hyclone, Thermo Scientific, USA), 2 mM L-glutamine, penicillin (100 U/ml) and streptomycin (100 μg/ml).

**Table 1:** Influenza A/H5N1 viruses used in this study

**Nomenclature**	**Clade/ Subclade**	**Host**	**Reciprocal HA titer**	**Reference/accession number**
A/duck/Thailand/144/2005*	1	duck	nd	Songserm et al., 2006 [38]
A/dog/Thailand-Suphanburi/KU-08/2004	1	dog	256	DQ530170-7
A/chicken/Thailand/NP-172/2006**	2.3.4	mouse	32	DQ999872-3

nd, not determined; * adamantane resistant; **mouse adapted, adamantine sensitive.

### Preparation of recombinant NP (rNP), recombinant M2 (rM2) and native M2 (nM2)

Coding sequences of NP (1,500 bp) and M2 (291 bp) were PCR amplified from A/duck/Thailand/144/2005 cDNA by using specific primers [21]. Each amplicon was cloned into pET-20b(+) vector (Novagen, USA) and introduced into BL21 (DE3) *E. coli*. Selected transformed *E. coli* clones were grown in LB medium containing 100 μg/ml ampicillin (LB-A) until the OD_600nm_ was 0.5. The *E. coli* cultures were induced with 0.5 mM IPTG to express the recombinant proteins. The bacterial lysates containing 6x-his-tagged-NP and -M2 were purified (Ni-NTA affinity resin, Thermo Science, USA). Each recombinant preparation was verified by 14% SDS-PAGE and Coomassie Brilliant Blue G-250 (CBB) staining, Western blot analysis and LC-MS/MS and protein content was determined.

MDCK cells infected for 24 h with mouse adapted A/chicken/Thailand/NP172/2006 at MOI 1 were harvested, washed with phosphate buffered saline, pH 7.4 (PBS) and subjected to ultrasonic disintegration in the buffer on ice. The homogenate was centrifuged and the supernatant (lysate) containing nM2 was kept at -20°C until use.

### Polyclonal antibodies (PAb) against rM2 and rNP

Animal experiments were approved by Veterinary Science-Animal Care and Use Committee (FVS-AUCU), Faculty of Veterinary Medicine, Kasetsart University, Thailand. ICR mice were immunized with either rM2 or rNP mixed with alum adjuvant [39]. Serum antibody titers against the homologous antigens were determined by indirect ELISA. Western blot analysis was used for determining antigenic specificities of the antibody preparations. Recombinant M2 and rNP were subjected to 14 and 12% SDS-PAGE, respectively. The separated components were electroblotted onto nitrocellulose membranes (NC). After unoccupied sites on the NC were blocked with 5% skim milk in PBS and washed, the NC were incubated with the mouse immune sera. Goat anti-mouse immunoglobulin-alkaline phosphatase conjugate and BCIP/NBT substrate were used for revealing the rM2/rNP-antibody reactive bands at ~17 and 60 kDa, respectively.

### Production and characterization of M2-specific human ScFv (HuScFv)

Phage clones displaying M2-bound HuScFv were selected from a human ScFv phage display library constructed previously [40]. Phage display library was added into an ELISA well coated with rM2. After incubation, unbound phages were removed by extensive washing. Log phase HB2151 *E. coli* suspension was added to the well containing the rM2 bound phages. The bacteria were spread on a selective agar plate and incubated overnight. Colonies on the agar were picked randomly and screened for the presence of HuScFv coding sequences (*huscfv*) by using pCANTAB5E specific primers [40]. Bacterial clones carrying *huscfv* were grown under IPTG induction for HuScFv expression [40]. The HuScFv in the *E. coli* lysates were tested for binding specificity to rM2 and nM2. Wells of ELISA plate (Costar, USA) were coated with either rM2 (1 μg in 100 μl of carbonate-bicarbonate buffer, pH 9.6) or the virus lysate containing nM2 (5 μg in 100 μl PBS buffer). BSA was used as control antigen. After blocking the antigen coated wells with 1% BSA, individual *E. coli* lysates containing HuScFv were added appropriately to the wells. Mouse monoclonal anti-E tag was added. Goat anti-mouse immunoglobulin-horseradish peroxidase (HRP) conjugate and ABTS substrate were used for color reaction. Antigen coated wells added with lysate of original HB2151 *E. coli* and buffer served as background control and blank, respectively. OD_405nm_ of the content each well was measured against blank. *E. coli* clones that the lysate reacted with the antigen and gave OD_405nm_ above the background control and two times higher than the BSA control were selected for further experiment.

Antigenic specificity of the HuScFv in *E. coli* lysates were also determined by Western blot analysis. Recombinant and native M2 were subjected separately to 14% SDS-PAGE and the separated components were blotted onto a nitrocellulose membrane (NC). After blocking the NC empty sites with 5% skim milk in PBS, the blotted NC was cut vertically into strips. The strips were incubated with the individual *E. coli* lysates containing HuScFv at 25°C for 2 h. The unbound materials were removed by washing with PBS containing 0.05% Tween-20 (PBST). The strips were reacted with mouse monoclonal anti-E-tag or mouse polyclonal antibody to rM2 and washed. Goat anti-mouse immunoglobulin-alkaline phosphatase (AP) conjugate and BCIPT/NBT substrate were used for revealing the antigen-antibody reactive bands.

Binding of the HuScFv to native M2 in the influenza virus infected cells were performed. MDCK cells infected with H5N1 virus at MOI 1 were cultured in a 24 well-plate at 37°C in 5% CO_2_ atmosphere for 24 h. The infected cells were washed and permeabilized with 1% Triton-X 100 in PBS for 20 min. After blocking with 3% fetal bovine serum, the permeabilized cells were added with HuScFv or PAb to M2 and kept at 25°C for 1 h. After washing, mouse monoclonal anti-His tag was added to the well containing cells that had been exposed to HuScFv and kept at 25°C for 1 h. Chicken anti-mouse immunoglobulin-Alexa Fluor^®^ 488 was added to all wells and kept at 25°C for 1 h. The cells were washed thoroughly, mounted and observed under a fluorescence microscope.

Restriction fragment length polymorphism (RFLP) of *huscfv* from individual *E. coli* clones was determined by using *Mva*I restriction enzyme digestion [41]. Digested DNA preparations were subjected to 14% polyacrylamide gel electrophoresis and ethidium bromide staining. Banding patterns of *huscfv* DNA of all clones were compared [41].

Immunoglobulin frameworks (FRs) and complementarity determining regions (CDRs) of the deduced amino acids of *huscfv* sequences were predicted using the International Immunogenetics information system (IMGT/V-QUEST) [35].

For large scale production of the HuScFv, the *huscfv* of *E. coli* clones of interest were subcloned into pET32c(+) *via* restriction endonuclease (*Hind*III and *Not*I) (Fermentas, Lithuania). The recombinant plasmids were introduced into BL21 (DE3) *E. coli*. Appropriate transformed *E. coli* clones were inoculated individually into 250 mL 2 × YT (Yeast Extract Tryptone) broth containing 100 μg/mL ampicillin and induced for HuScFv expression under 0.5 mM IPTG at 37°C with shaking for 4 h. Bacterial cells were harvested, sonicated in buffer (50 mM sodium phosphate, 8 M urea, and 300 mM NaCl, pH 7.0) and centrifuged at 12,000 × g, 4°C, 15 min. HuScFv in each lysate was purified (Talon® metal affinity resin, Takara, Japan). The HuScFv was dialyzed against PBS and filtrated through 0.45-μm membrane (Pall, USA) before use. Reactivity of the purified HuScFv to rM2 was verified by indirect ELISA and Western blot analysis using rM2 as the antigen and mouse monoclonal anti-6x-His antibody as the primary antibody for detecting the 6x-His tagged-HuScFv.

### HuScFv mediated interference of virus replication cycle

Influenza viruses were either added directly (experiment 1) or mixed with purified HuScFv (400 ng, ~9.64 × 10^12^ molecules) from individual *E. coli* clones at 25°C for 2 h before adding (experiment 2) to MDCK cell monolayer in tissue culture wells (MOI 0.1). Viruses exposed to rimantadine (0.8 μM, ~4.82 × 10^17^ molecules) and polyclonal antibody to M2 (PAb) (10 μg, ~4 × 10^13^ molecules) instead of the HuScFv were used as positive inhibition controls. The viruses in culture medium served as negative inhibition or infected cell control. The plates were incubated in 5% CO_2_ atmosphere, 37°C, 1 h to allow the virus entry. Extracellular viruses in each well were discarded; the cells were rinsed with plain DMEM and replenished with viral growth medium [DMEM containing 2% FBS, 2 mM L-glutamine, penicillin (100 U/mL), streptomycin (100 μg/mL)] containing the respective inhibitors/controls, i.e., 400 ng HuScFv, 10 μg PAb, 0.8 μM rimantadine or medium alone). After incubation for 15 h, the spent culture fluid of each well was collected and the cells were washed with PBS and harvested. Quantitative real-time RT-PCR was used for enumeration of the viruses in the culture fluids and inside the cells. Plaque (foci) assay was also performed for comparing intracellular viruses among treatments. Three independent experiments were done.

### Quantitative real-time RT PCR (qPCR)

Viral RNA was extracted from 500 μL of each culture supernatant by using viral nucleic acid extraction kit II (Geneaid, USA). The RNA was dissolved with 20 μL of DEPC-treated water. Total RNA was extracted from the virus infected cells by using total RNA purification kit (Jena Bioscience, Germany). The amount of total RNA in each preparation was measured by using NanoDrop ND-1000 Spectrophotometer (Thermo Scientific, USA) and kept at -70°C until use. In each qPCR reaction, either 2 μL of extracted RNA from the culture supernatant or 200 ng of total RNA from infected cells were subjected to viral RNA quantification using 1-step Brilliant II SYBR green qRT-PCR master mix kit (Agilent Technologies, USA). Each PCR master mix (12.5 μL) was prepared on ice with 2 × Brilliant II SYBR® Green QRT-PCR Master Mix, RT/RNase block enzyme, 200 nM of each primer specific to gene sequence coding for influenza A virus *M1*: forward 5′-CTTCTAACCGAGGTCGAATCGTA-3′ and reverse 5′-TCCATGAGAGCCTCGAGAT-3′, and RNA template. PCR was performed using Mx 3000PTM instrument (Stratagene, USA). The qPCR condition was: reverse transcription at 42°C for 1 h, initial denaturation at 95°C for 10 min and 40 cycles at 95°C for 1 min, 58°C for 1 min and 72°C for 1 min. The thermal profile used for dissociation curve analysis was: 95°C for 1 min then ramped down to 55°C (0.5°C/s) and ramped up to 95°C. A standard curve was constructed from Ct of ten-fold dilutions of the pET20b(+) carrying gene sequence coding for M1 (ranged from 1000 to 1 ng or 2.07 × 10^11^ to 2.07 × 10^8^ copies). Ct of each sample was expressed as log_2_ of RNA copies calculated from the standard curve.

### Plaque assay

Influenza virus plaques (foci) in the infected cells were revealed as described previously [42]. Infected cells in each tissue culture well were washed with PBS, fixed with cold absolute methanol at 25°C, 1 h, washed again, and permeabilized by using 0.3% Triton X-100 in PBS for 20 min. The cells were blocked by incubating with 3% FBS at 25°C, 1 h. After washing, mouse PAb to rNP (1:1,000) was added and incubated at 25°C, 1 h. Goat anti-mouse-AP (1:3,000) was added, incubated at 25°C for 1 h and the cells were equilibrated in 0.15 M Tris–HCl, pH 9.6, at 25°C for 15 min before adding with BCIP/NBT substrate for color development. When the purple foci appeared under inverted microscope the reaction was stopped by rinsing with distilled water. The foci in the MDCK cell monolayer were observed and counted under a light microscope.

### Phage mimotopes, HuScFv epitopes and validation of the phage mimotope

Ph.D.-12™ phage display peptide library (New England Biolabs, USA) was used to determine HuScFv bound phage mimotopes as described previously [41]. Mimotope peptide sequences were deduced from the phage DNA sequences by DNAMAN software version 4.15. The mimotopes were classified into mimotope types by using Phylogeny ClustalW [43] The sequences of each mimotope type was multiply aligned by Kalign with M2 linear sequences of drug sensitive and resistant A/H5N1 viruses (Accession numbers AY651385.1 for clade 1 and AB478035.1 for clade 2) for identification of M2 residues bound by the HuScFv (epitopes) [35].

Phage clones displaying the mimotopes were tested in competitive ELISA for determining their capacity in blocking the binding HuScFv to rM2 as described previously [35]. Mimotopic phages were propagated in ER2738 *E. coli* and the titers of the amplified phages were determined according to manufacturer’s instruction (New England Biolabs, USA). Various amounts (10^4^, 10^5^ and 10^6^ plaque forming unit; pfu) of the phages (50 μl) were mixed individually with fixed amount of HuScFv (5 μg in 50 μl) and incubated at 37°C for 1 h. The HuScFv mixed with irrelevant phage mimotope served as background inhibition controls and HuScFv in buffer were negative inhibition controls (maximum binding, 100%). After adding individual mixtures to the rM2 coated wells and incubated, all wells were washed and mouse monoclonal anti-E tag antibody, goat anti-mouse immunoglobulin-HRP conjugate and ABTS substrate were added, respectively, with washing between steps. OD_405nm_ of the content of each wells were determined against the background binding controls. The % ELISA inhibition was calculated:

$$\%\;\text{ELISA}\;\text{inhibition}=\left[\frac{\left({\mathrm{OD}}_{405\mathrm{nm}}\mathrm{of}\;\text{maximum}\;\text{binding}-{\mathrm{OD}}_{405\mathrm{nm}}\mathrm{of}\;\text{test}\right)}{\left({\mathrm{OD}}_{405\mathrm{nm}}\text{maximum}\;\text{binding}\right)}\right]$$

### Homology modelling and molecular docking to determine the regions and residues of tetrameric M2 ion channel bound by the HuScFv

Amino acid sequences similar to the rM2 and the HuScFv were identified by using a Discovery studio (DS) database. Three dimensional (3D) structures of the sequences that provided maximum identities were used as templates for homology modelling. The modelled structures were validated by PROCHECK analysis [44]. Complex structures between the modelled rM2 and HuScFv were predicted by using rigid bodies docking technique. The ZDOCK and RDOCK modules embedded on Discovery Studio program were used as tools for docking calculation and structural refinement, respectively. The modelled M2 was set as input receptor and the HuScFv as ligands. The dock complexes with the lowest RDOCK energy were determined for identification of the regions and residues of the tetrameric M2 ion channel interacting with the HuScFv.

### Multiple alignments of M2 sequences of various influenza A subtypes and H5N1 clades

The amino acid sequences of influenza A viruses including H1N1, H3N2, H5N1 (clades 0, 1, 2 and 7 which infect humans), H7N3, H7N9 and H9N2 were retrieved from the NCBI database (http://www.ncbi.nlm.nih.gov/protein). For the multiple sequence alignments, the program MAFFT version 7 was used. The alignments were verified following the algorithm of semi-homology. The verification concerned the possible genetic relationship between compared positions which were possible replacements by single transition/transversion.

### Statistical analysis

Means and standard deviations (SD) of log_2_ of M1 mRNA derived from triplicate cell culture wells of three independent experiments were used for comparison between tests and controls. *P* value < 0.05 of unpaired *t-*test was significantly different.

## Competing interests

The authors declare that they have no competing interests.

## Authors’ contributions

TP did most experiments, SM performed phage biopanning and HuScFv expressions, KT, prepared recombinant M2 and NP, PS supervised TP on qPCR, FD and JT, mimotope searching, TS isolated the H5N1 viruses and propagation, PT supervised TP on immunology methods, KB performed homology modeling and molecular docking. WC conceived the project and wrote the manuscript. All authors read and approved the final manuscript.

## Supplementary Material

Additional file 1: Figure S1 Binding of HuScFv of clones no. 2, 19, 23 and 27 and PAb to M2 in the MDCK cells infected with amantadine sensitive (NP-172) (blocks of middle column, respectively ) and resistant (KU08) (blocks of right column, respectively) H5N1 viruses. Non-infected cells reacted with HuScFv and PAb are shown in respective blocks of the left column. Click here for file

Additional file 2: Figure S2 Appearances of influenza virus foci in infected MDCK cells treated with HuScFv, PAb to M2 and rimantadine. Adamantane sensitive [A/chicken/Thailand/NP-172/2006 (H5N1 clade 2)] and resistant [A/dog/Thailand/KU-08/2004 (H5N1 clade 1) viruses were incubated with M2 specific-HuScFv before adding to MDCK cell monolayer. Viruses mixed with rimantadine and PAb to M2 were used as positive inhibition controls while viruses in culture medium served as negative inhibition (infected cell) controls. The cells were cultured in the medium containing respective HuScFv, rimantadine, PAb to M2 and medium alone for 15 h. Extracellular viruses were removed and the cells were washed before subjecting to immune-staining for virus plaques (foci). A and I: Uninfected MDCK cell monolayer; B and J: negative inhibition controls (MDCK cells infected with the viruses); C and K: MDCK cells infected with viruses exposed to PAb to M2; D and L: MDCK cells infected with rimantadine exposed viruses; E and M, F and N, G and O and H and P: MDCK cells infected with viruses exposed to HuScFv2, HuScFv19, HuScFv23 and HuScFv27, respectively. Click here for file

Additional file 3: Figure S3 Multiple alignments of the M2 amino acid sequences of various influenza A subtypes and clades of subtype H5N1. The peptides of M2 proteins which were binding sites of the HuScFv of clones no. 2, 19, 23 and 27 are highly conserved across A subtypes and H5N1 clades. Click here for file

## References

[B1] Nieva LJ Madan V Carrasco L Viroporins: structure and biological functions Nature Review 2012 10 563 574 10.1038/nrmicro2820 PMC7097105 22751485

[B2] Schnell JR Chou JJ Structure and mechanism of the M2 proton channel of influenza A virus Nature 2008 451 591 595 18235503 10.1038/nature06531 PMC3108054

[B3] Rossman SJ Lamb AR Influenza virus assembly and budding Virology 2011 411 2 229 236 21237476 10.1016/j.virol.2010.12.003 PMC3086653

[B4] Lamb AR Zebedee LS Richard DC Influenza virus M2 protein is an integral membrane protein expressed on the infected-cell surface Cell 1985 40 3 627 633 3882238 10.1016/0092-8674(85)90211-9

[B5] Zebedee LS Lamb AR Influenza A virus M2 protein Monoclonal antibody restriction of virus growth and detection of M2 in virion J Virol 1988 62 8 2762 2772 2455818 10.1128/jvi.62.8.2762-2772.1988 PMC253710

[B6] Helenius A Unpacking the incoming influenza virus Cell 1992 69 577 578 1375129 10.1016/0092-8674(92)90219-3

[B7] Ciampor F Bayley MP Nermut VM Hirst AME Sugrue JR Hay JA Evidence that the amantadine-induced, M2-mediated conversion of influenza A virus hemagglutinin to the low pH conformation occurs in an acid trans golgi compartment Virology 1992 188 14 24 1566569 10.1016/0042-6822(92)90730-d

[B8] Gannage M Dormann D Albrecht R Dengjel J Torossi T Matrix protein 2 of influenza A virus blocks autophagosome fusion with lysomes Cell 2009 6 367 380 10.1016/j.chom.2009.09.005 PMC2774833 19837376

[B9] Fiore EA Fry A Shay D Gubareva L Bresee SJ Uyeki MT Antiviral agents for the treatment and chemoprophylaxis of influenza MMWR 2011 60 RR01 1 24 21248682

[B10] Jefferson T Jones MA Doshi P Del Mar CB Henegham CJ Neuraminidase inhibitors for preventing and treating influenza in healthy adults and children (Review) The Cochrane library 2012 10 1 224 10.1002/14651858.CD008965.pub3 22258996

[B11] Sheu GT Fry MA Garten JR Deyde MV Shine T Bullion L Dual resistance to amantadine and oseltamivir among seasonal influenza A (H1N1) viruses: 2008–2010 J Infect Dis 2011 203 1 13 17 21148491 10.1093/infdis/jiq005 PMC3086447

[B12] Dolin R Reichman CR Madore PH Maynard R Linton NP Webber-Jones J A controlled trial of amantadine and rimantadine in the prophylaxis of influenza a infection N Engl J Med 1982 307 580 584 7050702 10.1056/NEJM198209023071002

[B13] Smirnov AV Lipatov SA Gitelman KA Class JE Osehuas EA Prevention and treatment of bronchopneumonia in mice caused by mouse-adapted variant of avian H5N2 influenza A virus using monoclonal antibody against conserved epitope in the HA stem region Arch Virol 2000 145 1733 1741 11003481 10.1007/s007050070088

[B14] Renegar BK Small AP Boykins GL Wright FP Role of IgA versus IgG in the control of influenza viral infection in the murine respiratory tract J Immunol 2004 173 1978 1986 15265932 10.4049/jimmunol.173.3.1978

[B15] Simmons PC Bernasconi LN Suguitan LA Jr Mills K Ward MJ Chau VN Prophylactic and therapeutic efficacy of human monoclonal antibodies against H5N1 influenza PLoS Med 2007 4 5 e178 17535101 10.1371/journal.pmed.0040178 PMC1880850

[B16] Luke TC Kilbane EM Jackson JL Hoffman SL Meta-analysis: convalescent blood products for Spanish influenza pneumonia: a future H5N1 treatment? Ann Intern Med 2006 145 8 599 609 16940336 10.7326/0003-4819-145-8-200610170-00139

[B17] Zhou B Zhong N Guan Y Treatmant with convalescent plasma for influenza A (H5N1) infection N Engl J Med 2007 357 14 1450 1451 17914053 10.1056/NEJMc070359

[B18] Maneewatch S Thanongsaksrikul J Songserm T Thueng-In K Kulkeaw K Human single-chain antibodies that neutralize homologous and heterologous strains and clades of influenza A virus Antivir Ther 2009 14 221 223 19430097

[B19] Treanor JJ Tierney LE Zebedee LS Lamb AR Murphy RB Passively transferred monoclonal antibody to the M2 protein inhibits influenza A virus replication in mice J Virol 1990 64 3 1375 1377 2304147 10.1128/jvi.64.3.1375-1377.1990 PMC249260

[B20] Kim R Toge T Changes in therapy for solid tumors: potential for overcoming drug resistance *in vivo* with molecular targeting agents Surg Today 2004 34 293 303 15052442 10.1007/s00595-003-2710-4

[B21] Theug-in K Maneewatch S Srimanote P Songserm T Tapchaisri P Heterosubtypic immunity to influenza mediated by liposome adjuvanted H5N1 recombinant protein vaccines Vaccines 2010 28 6765 6777 10.1016/j.vaccine.2010.07.065 20688037

[B22] Sharma M Yi M Dong H Qin H Peterson E Insight into the mechanism of the influenza A proton channel from a structure in a lipid bilayer Science 2010 330 6003 509 512 20966252 10.1126/science.1191750 PMC3384994

[B23] Achaya R Carnevale V Fiorin G Levine BG Polishchuk AL Structure and mechanism of proton transport through the transmembrane tetrameric M2 protein bundle of the influenza A virus Proc Natl Acad Sci 2010 107 34 15075 15080 20689043 10.1073/pnas.1007071107 PMC2930543

[B24] Stouffer LA Acharya R Salom A Levine SA Costanzo L Structural basis for the function and inhibition of an influenza virus proton channel Nature 2008 451 596 599 18235504 10.1038/nature06528 PMC3889492

[B25] Layne PS Monto SA Taubenberger KS Pandemic influenza: an inconvenient mutation Science 2009 323 5921 1560 1561 19299601 10.1126/science.323.5921.1560 PMC2778479

[B26] Balgi DA Wang J Cheng YHD Ma C Pfeifer AT Inhibitors of the influenza A virus M2 ion channel discovered using a high-throughput yeast growth restoration assay PLoS One 2013 8 2 e55271 23383318 10.1371/journal.pone.0055271 PMC3562233

[B27] Pielak MR Schnell RJ Chou JJ Mechanism of drug inhibition and drug resistance of influenza A M2 channel Proc Natl Acad Sci 2009 18 7379 7384 19383794 10.1073/pnas.0902548106 PMC2678642

[B28] Balannik V Wang J Ohigashi Y Jing X Magavern E Design and pharmacological characterization of inhibitors of amantadine-resistant mutants of the M2 ion channel of influenza A virus Biochemistry 2009 48 50 11872 11882 19905033 10.1021/bi9014488 PMC2794939

[B29] Du J Cross AT Zhou HX Recent progress in structure-based anti-influenza drug design Drug Discov Today 2012 17 1111 1120 22704956 10.1016/j.drudis.2012.06.002 PMC3459301

[B30] Carrasco L Modification of membrane permeability by animal viruses Adv Virus Res 1995 45 61 112 7793329 10.1016/S0065-3527(08)60058-5 PMC7131156

[B31] Gonzalez ME Carrasco L Viroporins FEBS Lett 2003 552 28 34 12972148 10.1016/s0014-5793(03)00780-4

[B32] Hout SD Vpu: A multifunctional protein that enhances the pathogenesis of human immunodeficiency virus type 1 Curr HIV Res 2004 2 255 270 15279589 10.2174/1570162043351246

[B33] Wang J Qui JX Soto C DeGrado WF Structural and dynamic mechanisms for the function and inhibition of the M2 proton channel from influenza A virus Curr Opin Struct Biol 2011 21 68 80 21247754 10.1016/j.sbi.2010.12.002 PMC3039100

[B34] Carrasco L Membrane leakiness after viral infection and a new approach to the development of antiviral agents Nature 1978 272 694 699 205795 10.1038/272694a0

[B35] Thueng-in K Thanongsaksrikul J Srimanote P Bangphoomi K Pougpair O Cell penetrable humanized-VH-V_**H**_H that inhibit RNA dependent RNA polymerase (NS5B) of HCV PLoS One 2012 7 11 e49254 23145135 10.1371/journal.pone.0049254 PMC3493538

[B36] World Health Organization WHO Manual on Animal Influenza Diagnosis and Surveillance. Department of Communicable Disease Surveillance and Response 2002 http://www.who.int/vaccine_research/diseases/influenza/WHO_manual_on_animal-diagnosis_and_surveillance_2002_5.pdf

[B37] Groves JI Reeves BM Sinclair HJ Lytic infection of permissive cells with human cytomegalovirus is regulated by an intrinsic ‘pre-immediated-early’ repression of viral gene expression mediated by histone post-translational modification J Gen Virol 2009 90 2364 2374 19515830 10.1099/vir.0.012526-0

[B38] Songserm T Jam-on R Sae-Heng N Meesak N Meemak N Hulse-Past D Domestic ducks and H5N1 influenza epidemic Thailand Emerg Infect Dis 2006 12 4 575 581 16704804 10.3201/eid1204.051614 PMC3294714

[B39] Coligan EJ Kruisbeek MA Margulies HD Shevach ME Strober W Current protocols in immunology Wiley 2012 7 1934 3671

[B40] Kulkeaw K Sakolvaree Y Srimanote P Tongtawe P Maneewatch S Human monoclonal ScFv neutralize lethal Thai cobra, *Naja kaothia*, neurotoxin J Proteomics 2009 72 270 282 19162253 10.1016/j.jprot.2008.12.007

[B41] Thathaisong U Maneewatch S Kulkeaw K Thueng-In K Poungpair O Srimanote P Human monoclonal single chain antibodies (HuScFv) that bind to the polymerase proteins of influenza a virus Asian Pac J Allergy Immunol 2008 26 1 23 35 18595527

[B42] Rowe T Abernathy RA Hu-Primmer J Detection of antibody to avian influenza A (H5N1) virus in human serum by using a combination of serologic assays J Clin Microbiol 1999 37 937 443 10074505 10.1128/jcm.37.4.937-943.1999 PMC88628

[B43] EMBL-EBT Home page EBI Tools: Clustaw2-Phylogeny http://www.ebi.ac.uk/Tools/phylogeny/clustaw2_phylogeny

[B44] Laskowski RA MacArthur MW Moss DS Thornton JM PROCHECK: a PROgram to CHECK the stereochemical quality of protein structures J Appl Crystallogr 1993 26 283 291

