# Supplementary material for: Human monoclonal ScFv that bind to different functional domains of M2 and inhibit H5N1 influenza virus replication
Source: Virol J. 2013 May 14;10:148. doi: 10.1186/1743-422X-10-148 (PMC3660209; doi:10.1186/1743-422X-10-148)
Supplement: Additional file 1: Figure S1 — Binding of HuScFv of clones no. 2, 19, 23 and 27 and PAb to M2 in the MDCK cells infected with amantadine sensitive (NP-172) (blocks of middle column, respectively ) and resistant (KU08) (blocks of right column, respectively) H5N1 viruses. Non-infected cells reacted with HuScFv and PAb are shown in respective blocks of the left column. [file 1743-422X-10-148-S1.docx]

**Supplemenatry Figure 1** Binding of HuScFv of clones no. 2, 19, 23 and 27 and PAb to M2 in the MDCK cells infected with amantadine sensitive (NP-172) (blocks of middle column, respectively ) and resistant (KU08) (blocks of right column, respectively) H5N1 viruses. Non-infected cells reacted with HuScFv and PAb are shown in respective blocks of the left column.
