# Supplementary material for: Human monoclonal ScFv that bind to different functional domains of M2 and inhibit H5N1 influenza virus replication
Source: Virol J. 2013 May 14;10:148. doi: 10.1186/1743-422X-10-148 (PMC3660209; doi:10.1186/1743-422X-10-148)
Supplement: Additional file 2: Figure S2 — Appearances of influenza virus foci in infected MDCK cells treated with HuScFv, PAb to M2 and rimantadine. Adamantane sensitive [A/chicken/Thailand/NP-172/2006 (H5N1 clade 2)] and resistant [A/dog/Thailand/KU-08/2004 (H5N1 clade 1) viruses were incubated with M2 specific-HuScFv before adding to MDCK cell monolayer. Viruses mixed with rimantadine and PAb to M2 were used as positive inhibition controls while viruses in culture medium served as negative inhibition (infected cell) controls. The cells were cultured in the medium containing respective HuScFv, rimantadine, PAb to M2 and medium alone for 15 h. Extracellular viruses were removed and the cells were washed before subjecting to immune-staining for virus plaques (foci). A and I: Uninfected MDCK cell monolayer; B and J: negative inhibition controls (MDCK cells infected with the viruses); C and K: MDCK cells infected with viruses exposed to PAb to M2; D and L: MDCK cells infected with rimantadine exposed viruses; E and M, F and N, G and O and H and P: MDCK cells infected with viruses exposed to HuScFv2, HuScFv19, HuScFv23 and HuScFv27, respectively. [file 1743-422X-10-148-S2.docx]

**A/chicken/Thailand/NP-172/2006 (H5N1 clade 2) A/dog/Thailand/KU-08/2004 (H5N1 clade 1)**

**Supplementary Figure 2** Appearances of influenza virus foci in infected MDCK cells treated with HuScFv, PAb to M2 and rimantadine. Adamantane sensitive [A/chicken/Thailand/NP-172/2006 (H5N1 clade 2)] and resistant [A/dog/Thailand/KU-08/2004 (H5N1 clade 1) viruses were incubated with M2 specific-HuScFv before adding to MDCK cell monolayer. Viruses mixed with rimantadine and PAb to M2 were used as positive inhibition controls while viruses in culture medium served as negative inhibition (infected cell) controls. The cells were cultured in the medium containing respective HuScFv, rimantadine, PAb to M2 and medium alone for 15 h. Extracellular viruses were removed and the cells were washed before subjecting to immune-staining for virus plaques (foci). A and I: Uninfected MDCK cell monolayer; B and J: negative inhibition controls (MDCK cells infected with the viruses); C and K: MDCK cells infected with viruses exposed to PAb to M2; D and L: MDCK cells infected with rimantadine exposed viruses; E and M, F and N, G and O and H and P: MDCK cells infected with viruses exposed to HuScFv2, HuScFv19, HuScFv23 and HuScFv27, respectively.
