# Supplementary material for: Human monoclonal ScFv that bind to different functional domains of M2 and inhibit H5N1 influenza virus replication
Source: Virol J. 2013 May 14;10:148. doi: 10.1186/1743-422X-10-148 (PMC3660209; doi:10.1186/1743-422X-10-148)
Supplement: Additional file 3: Figure S3 — Multiple alignments of the M2 amino acid sequences of various influenza A subtypes and clades of subtype H5N1. The peptides of M2 proteins which were binding sites of the HuScFv of clones no. 2, 19, 23 and 27 are highly conserved across A subtypes and H5N1 clades. [file 1743-422X-10-148-S3.docx]

**Supplementary Figure 3** Multiple alignments of the M2 amino acid sequences of various influenza A subtypes and clades of subtype H5N1. The peptides of M2 proteins which were binding sites of the HuScFv of clones no. 2, 19, 23 and 27 are highly conserved across A subtypes and H5N1 clades.
